# Potential Prognostic Relevance of Left-Ventricular Global Longitudinal Strain and of the Summation of the Mitral and Tricuspid Regurgitation Volume in Patients with Non-Ischemic Dilated Cardiomyopathy

**DOI:** 10.3390/jcdd10100410

**Published:** 2023-09-27

**Authors:** Karolina Mėlinytė-Ankudavičė, Eglė Ereminienė, Vaida Mizarienė, Gintarė Šakalytė, Jurgita Plisienė, Renaldas Jurkevičius

**Affiliations:** 1 Department of Cardiology, Medical Academy, Lithuanian University of Health Sciences, LT-44307 Kaunas, Lithuania; egle.ereminiene@lsmu.lt (E.E.); vaida.mizariene@lsmu.lt (V.M.); gintare.sakalyte@lsmu.lt (G.Š.); jurgita.plisiene@lsmu.lt (J.P.); renaldas.jurkevicius@lsmu.lt (R.J.); 2 Institute of Cardiology, Lithuanian University of Health Sciences, LT-50162 Kaunas, Lithuania

**Keywords:** heart failure, non-ischemic dilated cardiomyopathy, 2D echocardiography, early primary outcomes

## Abstract

Background: The aim of this pilot study was to determine the potential prognostic relevance of novel multidirectional myocardial and volumetric echocardiographic parameters in patients with non-ischemic dilated cardiomyopathy (NIDCM). Methods: Multidirectional myocardial parameters (longitudinal, radial, and circumferential left-ventricular (LV) strain using speckle tracking echocardiography) and a new volumetric parameter (the sum of the mitral and tricuspid regurgitation volume (mitral–tricuspid regurgitation volume) were assessed. The cardiovascular (CV) outcome was a composite of cardiac death and hospitalization for heart failure (HF) at 1 year. Results: Approximately 102 patients were included in this pilot study. The mean LV ejection fraction (LVEF) was 28.4 ± 8.9%. During a follow-up of 1 year, the CV outcome occurred in 39 patients (10 HF deaths, and 36 hospitalizations for HF). The LV global longitudinal systolic strain (GLS) and mitral–tricuspid regurgitation volume were the main parameters that were seen to be significantly altered in the comparison of patients with events vs. those without events (GLS (absolute values) 7.4 ± 2.7% vs. 10.3 ± 2.6%; mitral–tricuspid regurgitation volume 61.1 ± 20.4 mL vs. 40.9 ± 22.9 mL, respectively; *p*-value < 0.01). In line with these findings, in a multivariate continuous logistic regression analysis, the GLS and mitral–tricuspid regurgitation volume were the main parameters associated with worse CV outcomes (GLS: OR 0.77 (95%CI 0.65–0.92); mitral–tricuspid regurgitation volume OR 1.09 (95%CI 1.01–1.25)), whereas the radial and circumferential LV global strain and mitral regurgitation volume and tricuspid regurgitation volume were not linked to the CV outcome. Furthermore, in a receiver operating characteristic curve analysis, a GLS cutoff of <7.5% and mitral–tricuspid regurgitation volume > 60 mL were the identified values for the parameters associated with worse CV outcomes. Conclusions: The findings of this pilot study suggest that the GLS and a novel volumetric parameter (the sum of the mitral and tricuspid regurgitation volume) are linked to worse CV outcomes in patients with non-ischemic dilated cardiomyopathy. Hence, these promising results warrant further validation in larger studies.

## 1. Introduction

Dilated cardiomyopathy (DCM) is associated with LV or biventricular dilation and systolic dysfunction in the absence of abnormal loading conditions. This pathology is a frequent cause of HF and heart transplantation. DCM has a long sub-clinical period occurring with or without symptoms and myocardial changes, which increases the chance of a missed early diagnosis [1,2]. The progression of DCM involves various interactions between the cardiovascular system and neurohormonal factors that are related to the remodeling of the whole-heart myocardium [3]. Various factors affect the prognosis of DCM, such as age, gender, genetics, comorbidities, etc. [4] Studies on HF have been mostly focused on the assessment of the unidirectional function (i.e., longitudinal or circumferential) of the LV [5] and have less often focused on the other part of the heart [6,7]. Previous studies have shown that using multidirectional systolic parameters (i.e., the global systolic index (GSI) or longitudinal–circumferential index) may provide additional clinical value in assessing the global LV systolic function compared with measurements only of the LVEF [5]. Moreover, recent HF studies propose the use of the bivalvular (mitral–tricuspid) regurgitation volume because it reflects the global hemodynamic load and has a better relationship with CV outcomes, compared with single valvular lesions [8]. There is a lack of studies investigating whole-heart myocardial mechanics in certain diseases.

Previous results have shown that DCM is related to a poor prognosis with high mortality in the early and late periods of the disease [9,10]. Risk evaluation in NIDCM plays a crucial role in disease management, affecting treatment and prognosis. Although the disease is associated with high mortality, the prognosis has improved over the past few years due to optimal medical treatment and early diagnosis [11]. The diagnosis of DCM includes careful clinical assessment (personal and family history, physical examination, etc.) and second-line evaluation, which involves imaging, biopsy, and genetic testing [12]. Factors related to adverse events may help in detecting patients who need closer follow-up. There are many studies that show results about prognostic factors in HF [13,14] or NIDCM patients [11,15]. The evaluation of the risk factors for adverse outcomes within the early period for NIDCM patients is crucial to determining the most suitable subjects for heart transplantation or device implantation. In DCM, the most important predictors of prognosis are morphological, clinical, and hemodynamic parameters. However, current prediction models are supported by many variables, and there is a lack of data on simple and used-in-daily-practice predictors that are associated with DCM [16]. To our knowledge, previous studies have not evaluated the correlation between whole-heart myocardial function or morphometrics and early clinical outcomes in NIDCM. We aimed to detect the potential prognostic relevance of whole-heart myocardial deformation and morphometric parameters to early primary outcomes in patients with NIDCM and advanced HF.

## 2. Materials and Methods

### 2.1. Study Design

This was a single-center retrospective–prospective study involving 102 patients with NIDCM. The NIDCM diagnosis was defined according to the latest European Society of Cardiology (ESC) document [12]. The exclusion criteria for this study were ischemic coronary disease, primary valvular heart disease, chronic severe kidney disease (estimated glomerular filtration rate (eGFR) < 30 mL/min/1.73 m^2^), poor echocardiographic image quality, inflammatory (myocarditis, etc.) or infiltrative myocardial disease, tachycardia-induced HF (chronic or prolonged unknown duration of atrial fibrillation or atrial flutter), previous pulmonary embolism, peripartum cardiomyopathy, patients with an intra-cardiac defibrillator or cardiac resynchronization therapy, toxic damage (alcohol, drugs), and being under the age of 18. Ischemic coronary disease was excluded via angiography or computed tomography and was defined as the presence of a luminal reduction of ≥50% in the epicardial vessels [11,17], and a history of myocardial infarction or revascularization. Arterial hypertension was defined as the presence of an elevated systolic (>140 mm Hg) and/or diastolic (>90 mm Hg) blood pressure or the current use of antihypertensive drugs [18]. A patient was considered a smoker if he or she was currently smoking or had been a smoker in the past. Dyslipidemia was defined as the detection of any of the following criteria: serum total cholesterol ≥ 5.2 mmol/L, low-density lipoproteins > 2.6 mmol/L, triglycerides ≥ 1.7 mmol/L, or the current use of statin medication [19]. Diabetes mellitus was defined as the collective term for heterogeneous metabolic disorders whose main finding is chronic hyperglycemia [20].

Before enrollment, all patients underwent a detailed evaluation (including a detailed clinical, physical, and medical history), a laboratory test, an electrocardiogram (ECG), Holter monitoring, and 2D transthoracic echocardiography. A cardiac MRI was performed to clarify the specific pathology of the myocardium (inflammatory, infiltrative myocardial disease, etc.). In addition, patients were referred for genetic testing (next-generation sequencing of 231 genes coding regions related to inherited heart disorders was performed) and consulted by a geneticist. All participants gave written informed consent before enrollment. This study was approved by the local institutional ethics committee.

This study consisted of two phases:

- during the first phase, patients were enrolled, examined for the first time, and diagnosed with NIDCM (patients without chronic or WHF in their medical history and optimal medical therapy for HF).
- during the second phase, the early primary outcomes of a total of 102 patients with diagnosed NICDM were evaluated after a 1-year follow-up from diagnosis.

During follow-up, all patients were treated with an optimal HF treatment according to chronic HF guidelines [21]. Information about the presented adverse events was collected from medical records, via telephone calls (if the patient could not come to the hospital), or during hospital visits. The early primary outcomes were cardiac death and hospitalization for WHF at 1 year. WHF was defined according to current recommendations from the American College of Cardiology (i.e., patients admitted to hospital with decompensated HF requiring treatment with intravenous HF drugs) [22].

### 2.2. 2D Echocardiographic Data

Two-dimensional echocardiography was performed using the Philips “EPIQ 7” according to the European Association of Cardiovascular Imaging recommendations and the EACVI/ASE/Industry Task Force consensus documents [23,24]. The patients were studied by the same experienced echocardiographer in the left lateral decubitus position, and the first echocardiogram was performed during the first contact with the patient (within 24 h of the start of hospitalization or, in the case of an outpatient, during the first visit). The offline analysis of echocardiographic parameters was performed using TomTec Imaging Systems (Unterschleissheim, Germany) from archived cases.

The LV end-systolic and end-diastolic diameters were assessed from a parasternal LV long-axis view, and the volumes were analyzed using the biplane method of disk summation. The LVEF was assessed using Simpson’s biplane method [23]. For the assessment of the LVGLS, the apical four-chamber, two-chamber, and long-axis views were analyzed. For the evaluation of the global circumferential strain (GCS) or global radial strain (GRS), images were acquired at the basal, middle, and apical levels of the LV parasternal short-axis views [25].

The 2D STE was used for the measurement of the LV global systolic index (the average of the longitudinal, circumferential, and radial global systolic strain) and the longitudinal–circumferential systolic index (the average of the longitudinal and circumferential global systolic strain) [5].

The right-ventricular (RV) volumes were measured from the 4-chamber views. The RV free wall longitudinal strain (RVFWLS) was assessed from the three segments of the lateral wall (basal, mid-cavity, and apical). The global RV longitudinal strain (GRVLS) was analyzed from six segments of the RV free wall and the interventricular septum [24].

The right-atrial (RA) volume was assessed using the biplane method of disks in the apical 4-chamber view at end-systole. The LA size was analyzed at the end of the LV systole, and the LA volume (LAV) was measured in apical four- and two-chamber views using the disk summation algorithm [23]. The apical four-chamber view was used to automatically assess the reservoir, conduit, and contraction phases of the RA and LA [24].

The mitral–tricuspid regurgitation volume was presented as the sum of the mitral and tricuspid regurgitant volumes assessed using the proximal flow convergence method [8]. Three consecutive beats were averaged in patients with sinus rhythm and five consecutive beats in atrial fibrillation. The mitral–tricuspid regurgitation volume was estimated during the first contact with the patient before optimal medical treatment.

### 2.3. Intraobserver and Interobserver Variability

Approximately 25 patients were randomly selected to evaluate the intraobserver and interobserver variability, which showed a good agreement for the measurement of the LVGLS, with a small bias of 0.6 ± 2.9% and 0.5 ± 3.4%, respectively.

### 2.4. Statistical Analysis

The results were shown as mean ± standard deviation (SD) or as absolute numbers and percentages. A *p*-value of <0.05 was considered statistically significant. Analyses were performed using SPSS version 22 (IBM, Chicago, IL, USA). We divided our study population into two groups according to the presence of early primary outcomes (with or without the early primary outcomes). Student’s *t*-test was used to compare normally distributed variables and the Mann–Whitney U-test for abnormal. Binary logistic regression analysis was used to determine the potential predictors of early primary outcomes. The receiver operating characteristic (ROC) curve was plotted to evaluate the predictive value of the echocardiographic parameters to prognosticate early primary outcomes in patients with NIDCM. The cut-off value of the predictive model was defined as the point that yielded the maximum sum of sensitivity and specificity (Supplementary Materials).

## 3. Results

A total of 102 patients with NIDCM formed this study group, and the baseline clinical characteristics are summarized in Table 1. The mean age and gender balance did not differ between groups (*p* > 0.05). There were no statistically significant differences in the main risk factors (systolic blood pressure, dyslipidemia, arterial hypertension, etc.) between groups (*p* > 0.05). The presence of ventricular tachycardia was more common in the study group with early primary outcomes than in the group without (65.7% vs. 34.3%, *p* < 0.001). There was no statistically significant difference in atrial fibrillation (*p* > 0.005). During the first contact, the indicated drugs were usually used for arterial hypertension, alone or in combination therapy. The presence of early primary outcomes was not related to pathogenic variants in DCM. However, a large proportion of patients in this group refused genetic testing. Most of the patients had no stenosis in their coronary arteries. There was a statistically significant difference in brain natriuretic peptide concentrations between groups (*p* = 0.006). The early primary outcomes were determined in 39 patients (10 HF deaths, and 36 hospitalizations for WHF).

The data from the cardiac 2D echocardiography are shown in Table 2. The patients with a worse prognosis had more dilation in both their ventricles and atria; however, a statistically significant difference was presented only in the LV end-systolic diameter index (28.9 ± 5.1 vs. 26.7 ± 3.9, *p* = 0.022), the LV end-diastolic diameter index (34.1 ± 4.4 vs. 31.1 ± 3.4, *p* = 0.020), and the LA volume index (66.3 ± 40.6 vs. 50.2 ± 16.9, *p* = 0.027). The higher mitral–tricuspid regurgitation volume and worse LV GLS were found in the group with early primary outcomes (*p* = 0.003 and *p* = 0.001, respectively). The LV GRS and LV GCS did not correlate with a worse prognosis. The LASr function was worse in the study group with events (*p* < 0.05). The GSI and longitudinal–circumferential systolic index were reduced in both study groups; however, there was no statistically significant difference between these groups.

Only patients with an LVEF < 40% were included in the univariate and multivariate logistic regression analysis to determine the potential predictors of adverse events. The results in Table 3 show that the LV GLS and mitral–tricuspid regurgitation volume were independent predictors of a worse prognosis such as cardiac death or hospitalization for WHF after 1 year (*p* < 0.05).

**Figure 1:**
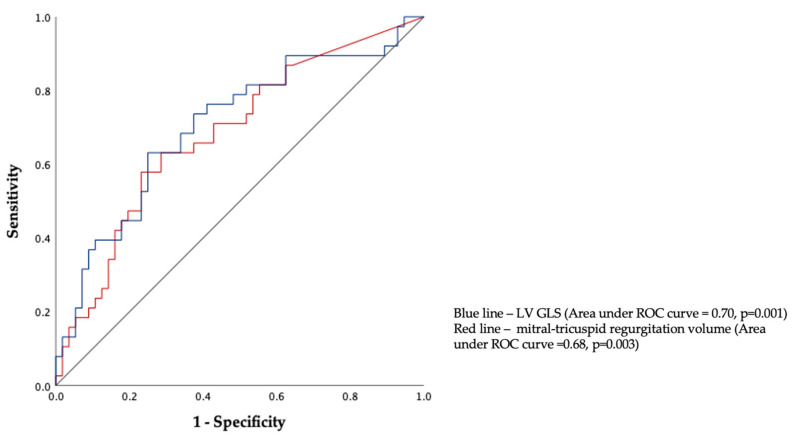
The area under the curve of the model in predicting early primary outcomes in patients with NIDCM. ROC—receiver operating characteristic; LV GLS—left-ventricular global longitudinal strain; red line—mitral–tricuspid regurgitation volume; green line—reference line.

According to the ROC analysis, patients with NIDCM with an LV GLS below −7.5% had a higher risk of primary adverse events than patients with an LV GLS > −7.5% (sensitivity of 85% and specificity of 84%). The cut-off value for the mitral–tricuspid regurgitation volume for the detection of a worse prognosis was 60 mL, with a sensitivity of 85% and a specificity of 87% (Figure 1).

## 4. Discussion

In this study, we evaluated the early primary outcomes of NIDCM patients with moderate-to-severe LV systolic dysfunction according to the 2D STE parameters and morphometrics of both the atria and ventricles. LV GLS and mitral–tricuspid regurgitation volume were the main independent prognostic factors to predict at least one of the following outcomes: cardiac death or hospitalization for WHF at 1 year. Patients with an LV GLS < −7.5% and mitral–tricuspid regurgitation volume values > 60 mL were at maximal risk for early adverse events.

The determination of the prognostic factors in HF plays a significant role in clinical treatment decisions and helps us to find the most suitable parameters to evaluate the clinical condition in the follow-up phase. Cardiac imaging methods such as cardiac magnetic resonance (CMR) or echocardiography can carefully assess the myocardial structure and function and have an important significance in the prognostic evaluation of patients with DCM [26]. As CMR is not always available, and because of patients frequently having implanted devices, it is not always possible to perform it. In this case, 2D echocardiography is considered the gold standard in clinical practice. Based on this, we performed STE to analyze the whole-heart myocardial mechanics. 

There are previous studies that have analyzed prognostic factors in DCM. In one large Swedish HF registry, the same primary outcomes were analyzed as in our study [27]. It was noticed that significant relationships with worse outcomes during all periods were found for age, a greater class of NYHA, a lower LVEF, and treatment with loop diuretics. However, this study was heterogenic and composed of various origins of DCM. Moreover, there was no assessment of the whole-heart myocardial mechanics and the impact of their changes on the outcome.

The results of many studies have shown that the LVEF is related to the prognosis of HF. A significant reduction in LVEF is associated with an increased risk of cardiovascular or all-cause mortality, HF hospitalization, or heart transplantation [28,29,30,31]. However, in clinical cases with advanced HF in patients with NIDCM, the degree of impaired LV systolic function loses its ability to predict survival [32]. Newer echocardiographic techniques, including STE, have been approved as being able to help find early-phase DCM and be used as a prognostic factor for mortality [28]. The analysis from previous studies has suggested that the global LV systolic function, using multidirectional parameters such as the global systolic index and the longitudinal–circumferential index, has advantages over the LVEF because it helps to assess the true global contractile function of the LV [5]. However, our results revealed no significant difference in these systolic parameters between the study groups. The results from many recent studies have shown that the assessment of the LV GLS might be a better independent prognostic factor in patients with HF [33,34]. It was detected that the global longitudinal strain detected via another modern method, CMR feature tracking, had an important role in prognosing mortality in a multicenter population of patients with DCM [35]. Therefore, with the matching of the echocardiographic and CMR results, we can use more accessible and simple examination methods in clinical practice, such as 2D STE. Our results showed a moderately strong LV GLS relationship with worse outcomes in patients with NIDCM, confirming previous results.

In DCM, functional mitral and tricuspid regurgitation are related to insufficient leaflet coaptation. The quantitative evaluation of the regurgitation severity can add important value to a patient’s risk stratification because the lesion severity is related to the outcomes [36,37]. The results of previous studies showed a significant impact of isolated valvular lesions on adverse outcomes [38]. However, recent research has shown that bivalvular functional regurgitation is related to a more rapid HF progression and has significant impacts on mortality. The impact of bivalvular functional regurgitation can be explained by volume overload that effects the eccentric chamber remodeling and additional downstream pressure [8]. To the author’s knowledge, no studies have evaluated the significance of the mitral–tricuspid regurgitation volume for outcomes in NIDCM. We have found that the assessment of the mitral–tricuspid regurgitation volume with LV GLS can help to prognosticate early adverse cardiovascular events in the population with NIDCM.

For many years, the focus has been on the evaluation of the LV, while the RV has been neglected. Advances in cardiac imaging have enabled a better evaluation of the RV, highlighting the importance of biventricular evaluation in DCM patients. The adverse remodeling of the RV has an important role in HF development, and RV systolic dysfunction is more common in NIDCM than in ischemic DCM [39]. Previous studies evaluated the prognostic role of RV function in NIDCM [40,41,42]. In contrast to our study, the influence of whole-heart mechanics on the disease prognosis was not analyzed. Gulati et al. reported that RV systolic dysfunction independently predicts adverse HF outcomes in DCM. However, this study assessed only the RVEF, without analysis of myocardial strain parameters. Moreover, this study population consisted of various phenotypic severities, in contrast to our study [40]. Liu T. et al. found an important prognostic value of the RVGLS to predict cardiovascular death and cardiac transplantation in NIDCM [32]. In our study, we did not find a significant correlation between a worse prognosis and changes in the mechanics or morphometrics of the right side of the heart.

### Clinical Perspectives

The assessment of whole-heart myocardial mechanics and morphometrics revealed that the mitral–tricuspid regurgitation volume, together with the LV GLS, can add prognostic information to the evaluation of patients in the early period of DCM, after prescribed optimal HF treatment. To prove these results, it is necessary to repeat the study or expand the sample size as, with larger cohorts, a stronger association with whole-myocardial mechanics could be detected.

## 5. Limitation

This study has some limitations. This is a single-center result and do not necessarily represent all patients with NIDCM. Given the small sample size of the study, a further, larger study should be used to validate the findings of this study.

## 6. Conclusions

The findings of this pilot study suggest that the GLS and a novel volumetric parameter (the sum of the mitral and tricuspid regurgitation volume) are linked to worse CV outcomes in patients with non-ischemic dilated cardiomyopathy. Hence, these promising results warrant further validation in larger studies.

## Figures and Tables

**Table 1:** The baseline characteristics for NIDCM patients with and without early primary outcomes.

Variables	Patients with Early Primary Outcomes *n* = 39	Patients without Early Primary Outcomes *n* = 63	*p*-Value
Age, y	48.5 ± 11.7	49.8 ± 10.0	0.574
Males, *n* (%)	26 (36.1)	46 (63.9)	0.510
BSA, m^2^	1.9 ± 0.2	2.0 ± 0.2	0.066
Heart rate, beat/min	81.3 ± 17.8	78.8 ± 15.8	0.467
Systolic blood pressure, mmHg	123.0 ± 14.0	127.5 ± 12.6	0.104
Dyslipidemia, *n* (%)	16 (40.0)	24 (60.0)	0.837
Arterial hypertension, *n* (%)	24 (39.3)	37 (60.7)	0.510
Smoking, *n* (%)	19 (45.2)	23 (54.8)	0.302
Diabetes mellitus, *n* (%)	4 (57.1)	3 (42.9)	0.425
Chronic kidney disease, *n* (%)	5 (55.6)	4 (44.4)	0.302
Genetic analysis: positive, *n* (%) Uncertain significance, *n* (%) Refused the genetic test, *n* (%)	5 (20.0) 5 (41.7) 11 (91.7)	20 (80.0) 7 (58.3) 1 (8.3)	<0.001
Pharmacotherapy (at baseline), *n* (%)			
ACE-I/ARB	11 (35.4)	20 (64.5)	0.742
Betablocker	9 (42.8)	12 (57.1)	0.381
CCB	5 (41.6)	7 (58.3)	0.402
Aldosterone antagonist	2 (40)	3 (60)	0.453
Statins	3 (37.5)	5 (62.5)	0.291
VT, *n* (%)	23 (65.7)	12 (34.3)	<0.001
Atrial fibrillation, *n* (%)	21 (50.0)	21 (50.0)	1.000
LBBB, *n* (%)	19 (43.2)	25 (56.8)	0.419
Prevalence of CA stenosis Without any CA stenosis, *n* (%) CA stenosis <50%, *n* (%)	24 (31.6) 15 (57.7)	52 (68.4) 11 (42.3)	*p* > 0.05
QRS duration, ms	125.5 ± 31.2	118.1 ± 27.6	0.226
NYHA class III-IV, *n* (%)	45 (62.3)	30 (37.7)	0.057
6MWT (<300 m), *n* (%)	10 (66.7)	5 (33.3)	0.602
Hs-CRP	3.0 ± 1.5	3.1 ± 1.6	0.854
BNP, ng/L	1812.1 ± 844.3	822.7 ± 425.6	0.006
Heart failure death, *n* (%)	10 (25.6)	-	-
Hospitalization for HF worsening at 1 year, *n* (%)	36 (92.3)	-	-

BSA—body surface area; ACE-I—angiotensin-converting enzyme inhibitor; ARB—angiotensin receptor blocker; CCB—calcium channel blocker; VT—ventricular tachycardia; LBBB—left bundle branch block; HF—heart failure; CA—coronary artery; NYHA—New York Heart Association; 6MWT—6 min walk test; Hs-CRP—high-sensitivity C-reactive protein; BNP—brain natriuretic peptide.

**Table 2:** The two-dimensional echocardiographic parameters in NIDCM patients with and without early primary outcomes.

Variables	Patients with Early Primary Outcomes *n* = 39	Patients without Early Primary Outcomes *n* = 63	*p*-Value
LVESD, mm	57.8 ± 9.0	55.1 ± 7.2	0.118
LVESDi, mm/m^2^	29.1 ± 5.1	26.7 ± 3.9	0.013
LVEDD, mm	66.1 ± 7.0	64.2 ± 5.8	0.128
LVEDDi, mm/m^2^	34.1 ± 4.4	31.1 ± 3.4	0.009
LAV, mL	134.3 ± 89.3	104. ± 36.6	0.064
LAVi, mL/m^2^	66.3 ± 40.6	50.2 ± 16.9	0.027
RAV, mL	81.6 ± 27.4	78.9 ± 23.1	0.585
RAVi, mL/m^2^	40.5 ± 10.3	38.1 ± 10.2	0.266
MRV, mL	33.3 ± 14.1	24.3 ± 18.0	0.005
TRV, mL	27.8 ± 14.7	16.6 ± 15.7	0.002
Mitral–tricuspid regurgitation volume, mL	61.1 ± 20.4	40.9 ± 22.9	0.003
LVEDV, mL	233.4 ± 75.2	230.7 ± 70.0	0.865
LVEDVi, mL/m^2^	117.4 ± 39.6	112.3 ± 35.3	0.526
LVESV, mL	173.8± 68.6	156.8 ± 59.5	0.219
LVESVi, mL/m^2^	90.0 ± 39.2	79.5 ± 36.1	0.198
RVEDVi, mL	76.8 ± 32.4	69.5 ± 26.8	0.069
RVESVi, mL	49.7 ± 26.4	42.1 ± 17.0	0.105
LV GLS, %	−7.4 ± 2.7	−10.3 ± 2.6	0.001
LV GCS, %	−12.2 ± 5.6	−14.7 ± 6.0	0.097
LV GRS, %	18.1 ± 9.2	21.6 ± 9.3	0.249
GSI, %	12.8 ± 4.5	12.7 ± 5.4	0.929
Longitudinal–circumferential systolic index, %	9.9 ± 3.7	9.7 ± 4.5	0.826
LVEF, %	27.3 ± 9.6	29.1 ± 8.5	0.356
RVFWLS, %	−17.7 ± 3.1	−18.5 ± 2.0	0.257
RVEF, %	37.3 ± 10.1	42.5 ± 7.9	0.099
RVGLS, %	−10.5 ± 3.7	−12.9 ± 3.2	0.053
FAC, %	30.9 ± 6.6	31.7 ± 5.5	0.868
LAScd, %	−12.1 ± 5.0	−13.7 ± 4.3	0.551
LASr, %	21.7 ± 4.1	24.8 ± 4.0	0.047
LASct, %	−9.3 ± 3.6	−10.3 ± 3.4	0.322
RAScd, %	−14.3 ± 5.8	−16.5 ± 5.1	0.065
RASr, %	28.9 ± 5.8	29.1 ± 6.5	0.875
RASct, %	−12.2 ± 5.2	−12.5 ± 6.1	0.771

LV—left-ventricular; LVESD—left-ventricular end-systolic diameter; LVESDi—left-ventricular end-systolic diameter index; LVEDD—left-ventricular end-diastolic diameter; LVEDDi—left-ventricular end-diastolic diameter index; LVEDV—left-ventricular end-diastolic volume; LVEDVi—left-ventricular end-diastolic volume index; LVESV—left-ventricular end-systolic volume; LVESVi—left-ventricular end-systolic volume index; GLS—global longitudinal strain; GCS—global circumferential strain; GRS—global radial strain; GSI—global systolic index; LVEF—left-ventricular ejection fraction; RVEDVi—right-ventricular end-diastolic volume index; RVESVi—right-ventricular end-systolic volume index; RVFWLS—right-ventricular free wall longitudinal strain; RVGLS—right-ventricular global longitudinal strain; RVEF—right-ventricular ejection fraction; FAC—fractional area change; LAV—left-atrial volume; LAVi—left-atrial volume index; RAV—right-atrial volume; LASr—left-atrial strain during the reservoir phase; LAScd—left-atrial strain during the conduit phase; LASct—left-atrial strain during the contraction phase; RAScd—right-atrial strain during the conduit phase; RASct—right-atrial strain during the contraction phase; RASr—right-atrial strain during the reservoir phase.

**Table 3:** Associations between the 2D echocardiographic parameters and the presence of early primary outcomes in the univariate and multivariate logistic regression analysis.

Parameter	Univariate Analysis			Multivariate Analysis		
	OR	95% CI	*p*	OR	95% CI	*p*
LV GLS, %	0.876	0.855–0.998	0.034	0.778	0.650–0.923	0.034
LASr, %	1.005	0.955–1.057	0.858	-	-	-
RVGLS, %	0.986	0.890–1.092	0.780	-	-	-
RVFWLS, %	0.979	0.881–1.089	0.701	-	-	-
LVEF, %	1.061	0.998–1.128	0.060	-	-	-
LAVi, mL/m^2^	0.976	0.955–0.998	0.034	0.971	0.932–1.012	0.161
TAPSE, mm	1.150	0.976–1.355	0.094	-	-	-
Mitral–tricuspid regurgitation volume, mL	0.976	0.955–0.998	0.034	1.098	1.012–1.295	0.008
MRV, mL	0.966	0.938–0.996	0.026	1.064	0.984–1.151	0.120
TRV, mL	0.971	0.942–1.001	0.061	-	-	-

LV—left-ventricular; GLS—global longitudinal strain; LASr—left-atrial strain during the reservoir phase; RVGLS—right-ventricular global longitudinal strain; RVFWLS—right-ventricular free wall longitudinal strain; LVEF—left-ventricular ejection fraction; LAVi—left-atrial volume index; TAPSE—tricuspid annular plane systolic excursion; mitral–tricuspid regurgitation volume; MRV—mitral regurgitation volume; TRV—tricuspid regurgitation volume; CI—confidence interval; OR—odds ratio.

## Data Availability

Not applicable.

## Supplementary Materials

The following supporting information can be downloaded at: https://www.mdpi.com/article/10.3390/jcdd10100410/s1, Table S1: Correlation analysis of early primary outcomes and whole-heart myocardial mechanics or morphometrics; Table S2: Binary logistic regression analysis for the 2D echocardiographic parameters related to the presence of early primary outcomes.
