# Supplementary material for: Potential Prognostic Relevance of Left-Ventricular Global Longitudinal Strain and of the Summation of the Mitral and Tricuspid Regurgitation Volume in Patients with Non-Ischemic Dilated Cardiomyopathy
Source: J Cardiovasc Dev Dis. 2023 Sep 27;10(10):410. doi: 10.3390/jcdd10100410 (PMC10606992; doi:10.3390/jcdd10100410)
Supplement: Supplementary file 1 [file jcdd-10-00410-s001.zip › jcdd-2474954-supplementary.pdf]

Journal of
Cardiovascular
Development and Disease

MDPI

Table S1. Correlation analysis of early primary outcomes and whole-heart myocardial mechanics or morphometrics.

	Early outcomes	
	**rs**	**p**
LVESDi, mm/m^2	-0.369	0.008
LVEDDi, mm/m^2	-0.378	0.006
LAVi, mm/m^2	-0.265	0.010
RAVi, mm/m^2	-0.109	0.296
GRV, ml	- 0.501	<0.001
LVEDVi, ml/m^2	- 0.068	0.515
LVESVi, ml/m^2	-0.137	0.189
RVEDVi, ml	- 0.123	0.239
RVESVi, ml	-0.173	0.096
LVGLS, %	-0.530	<0.001
LVGCS, %	-0.121	0.244
LVGRS, %	0.083	0.420
LVEF, %	0.195	0.342
GSI	-0.109	0.932
Longitudinal-circumferential index	-0.210	0.833
RVEF, %	0.179	0.081
RV GLS, %	-0.117	0.254
LAScd, %	-0.164	0.536
LASr, %	0.369	0.018
LASct, %	-0.114	0.270
RAScd, %	-0.196	0.056
RASr, %	0.116	0.379
RASct, %	-0.129	0.479

LVESDi – left ventricular end-systolic diameter index; LVEDDi – left ventricular end-diastolic diameter index; LVEDVi – left ventricular end-diastolic volume index; LVESVi – left ventricular end-systolic volume index; LVGLS – left ventricular global longitudinal strain; LVGCS – left ventricular global circumferential strain; LVGRS – left ventricular global radial strain; LVEF – left ventricular ejection fraction; RVEDVi – right ventricular end-diastolic volume index; RVESVi – right ventricular end-systolic volume index; RVGLS – right ventricular global longitudinal strain; GSI – global systolic index; RVEF – right ventricular ejection fraction; LAVi – left atrial volume index; RAVi – right atrial volume index; LASr – left atrial strain during reservoir phase; LAScd – left atrial strain during conduit phase; LASct – left atrial strain during contraction phase; RAScd – right atrial strain during conduit phase; RASct – right atrial strain during contraction phase; RASr – right atrial strain during reservoir phase.

Table S2. Binary logistic regression analysis for the 2D echocardiographic parameters related to the presence of early primary outcomes.

**Parameter**	**OR**	**95% CI**	***p***
LVGLS, %	0.770	0.646-0.918	0.004
GRV, ml	1.098	1.010-1.296	0.011

LVGLS – left ventricular global longitudinal strain; GRV – global regurgitation volume; CI – confidence interval; OR – odds ratio.
